# Cytokine profiles, blood parasite load and clinical features of visceral leishmaniasis in West Pokot County, Kenya

**DOI:** 10.1017/S0031182024000751

**Published:** 2024-06

**Authors:** Norbert van Dijk, Jane Carter, David Kiptanui, Elena Pinelli, Henk Schallig

**Affiliations:** 1 Department of Medical Microbiology and Infection Prevention, Laboratory for Experimental Parasitology, Amsterdam University Medical Centre, Amsterdam, The Netherlands; 2 Infectious Diseases Programme, Amsterdam Institute for Immunology and Infectious Diseases, Amsterdam, The Netherlands; 3 Regional Laboratory Programme, Amref Health Africa, Nairobi, Kenya; 4 Kacheliba Sub-County Hospital, Kacheliba, Kenya; 5 Centre for Infectious Disease Control, National Institute for Public Health and the Environment (RIVM), Utrecht, The Netherlands

**Keywords:** cytokines, haematology, Kenya, parasite load, visceral leishmaniasis

## Abstract

Visceral leishmaniasis (VL) is a severe infectious disease caused by protozoan parasites of the *Leishmania donovani* complex. Blood cytokine concentrations in VL patients can inform us about underlying immunopathogenesis and may serve as a biomarker for treatment effectiveness. However, cytokine levels have not yet been studied in VL patients from Kenya, where case load is high. This study measured the serum cytokine profile, blood parasite load and clinical and haematological features of VL patients from West Pokot County, Kenya, over the course of treatment with sodium stibogluconate and paromomycin (SSG-PM). VL patients recruited at the hospital presented with splenomegaly and weight loss, and frequently had pancytopenia and anaemia. Median *Leishmania* parasite load in blood, determined with real-time polymerase chain reaction, was 2.6 × 10^4^ parasite equivalents mL^−1^. Compared to endemic healthy controls, serum interferon gamma (IFN-*γ*), interleukin 5 (IL-5), IL-6, IL-10, IL-12p70, IL-17A and IL-27 were significantly elevated in untreated VL patients. Severe VL was associated with higher IL-10 and lower IFN-*γ* levels. After 17 daily injections with SSG-PM, disease symptoms disappeared, leukocyte and thrombocyte counts significantly increased, and blood parasite load decreased to undetectable levels in all VL patients. There was a significant decrease in IL-10 and IL-6, whereas IL-17A levels increased; the remaining cytokines showed no significant concentration change during treatment. In conclusion, the results suggest that SSG-PM treatment of VL patients from West Pokot was effective. Moreover, both inflammatory and regulatory immune responses appeared to decrease during treatment, although the increase in IL-17A could reflect a partial continuation of immune activation.

## Graphical abstract

**Figure:**
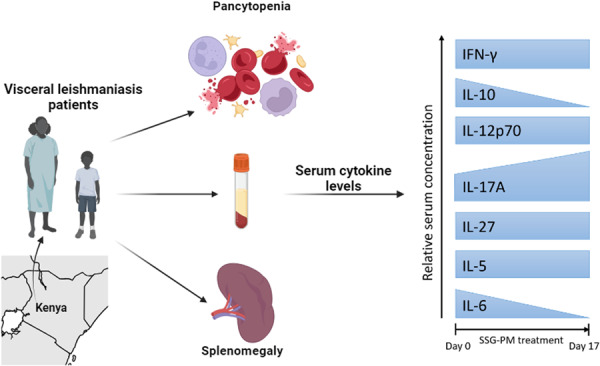

## Introduction

Visceral leishmaniasis (VL) is a serious parasitic disease caused by an infection with the protozoa *Leishmania donovani* or *Leishmania infantum*. Humans are infected with these parasites through the bite of an infectious female sandfly (*Phlebotomus* species). Inside the human host, the amastigote form of the parasite infects macrophages and other mononuclear phagocytes, particularly in the spleen, liver, bone marrow and lymph nodes (Costa *et al*., 2023). After a protracted incubation period, VL patients present with progressively severe disease characterized by chronic fever, splenomegaly, weight loss and pancytopenia. If left untreated, VL will be fatal in over 95% of cases (World Health Organization, 2023). A major part of the global VL burden occurs in the Horn of Africa, where several countries face endemic transmission of *L. donovani* (Ruiz-Postigo *et al*., 2021).

It is generally accepted that effective control of *Leishmania* infection is dependent on type 1-mediated immunity, whereby interferon gamma (IFN-*γ*) activates intracellular parasite-killing mechanisms in infected macrophages. However, *Leishmania* species significantly modulate this immune response, interfering with IFN-*γ* production and effector mechanisms by stimulating the secretion of the regulatory cytokine interleukin 10 (IL-10) (Rodrigues *et al*., 2016). As such, cytokines fulfil a pivotal role in the orchestration of VL immunology. Previous studies have shown that VL patients exhibit increased circulatory levels of both pro-inflammatory cytokines such as IFN-*γ*, IL-12p70, IL-6 and tumour necrosis factor alpha (TNF-*α*) as well as regulatory IL-10 and IL-27 (Ansari *et al*., 2006, 2011; van den Bogaart *et al*., 2014; Dos Santos *et al*., 2016). Furthermore, there has been increasing attention to the role of the T helper-17 (Th17) response, as elevated IL-17A and IL-22 have been linked to asymptomatic *L. donovani* infection (Pitta *et al*., 2009).

Describing serum cytokine levels can contribute to our understanding of VL immunopathogenesis and processes that lead to more severe disease and mortality (Costa *et al*., 2012; Dos Santos *et al*., 2016). In addition, cytokines could potentially be used as a prognostic marker for treatment efficacy and the probability of VL relapse (Kip *et al*., 2015). Given the wide geographical distribution of VL across different continents, it is likely that cytokine responses in VL are partially influenced by differences in patient populations and *Leishmania* species and strains (Duthie *et al*., 2014). There have been several studies in Brazil, the Indian sub-continent, Ethiopia and Sudan which investigated cytokine profiles of VL patients and, in some cases, their association with disease severity and treatment outcome (Hailu *et al*., 2005; Ansari *et al*., 2006; Duthie *et al*., 2014; van den Bogaart *et al*., 2014; Dos Santos *et al*., 2016). However, blood cytokine levels in VL have not been described in Kenya, even though this is currently 1 of the 6 countries with the highest VL case load in the world (Ruiz-Postigo *et al*., 2021; Kenya Ministry of Health, 2022). Moreover, changes in parasite load and cytokine concentrations in blood have not been investigated so far in VL patients treated with sodium stibogluconate and paromomycin (SSG-PM), which is the current first-line treatment for VL in eastern Africa (World Health Organization, 2010; Kenya Ministry of Health, 2017). To improve VL case management and care in Kenya, a better understanding of VL immunology and its associations with disease presentation and resolution upon SSG-PM treatment is warranted. This study in West Pokot County, Kenya, aimed to measure the serum cytokine profile and blood parasite load of VL patients before, during and after treatment with SSG-PM, and to investigate the association of these parameters with the patients' clinical and haematological features.

## Materials and methods

### Study site and study groups

Participants of this study were enrolled in the context of the cohort study component of the LEISHMAL project (registered at ISRCTN Registry, ref. 15023306) (van Dijk *et al*., 2023). VL patients were recruited in 2022 at Kacheliba Sub-County Hospital in West Pokot County, Kenya. This area is highly endemic for *L. donovani* transmission and Kacheliba Hospital is the regional reference centre for VL diagnosis and treatment (Kenya Ministry of Health, 2021). Individuals attending this hospital with symptoms of VL, including abdominal swelling, chronic fever and weight loss, were diagnosed according to the national guidelines by testing finger prick blood for anti-*Leishmania* antibodies using an IT LEISH (Bio-Rad, Hercules, USA) rK39 immunochromatographic test (ICT) (Kenya Ministry of Health, 2017). Positive cases above the age of 6 years, with no history of previous VL infection and negative for malaria as determined by blood film microscopy, were eligible to participate. Children younger than 6 years were excluded due to the burden of repetitive blood sampling involved in the study, and the generally immature status of their immune system which could bias the study findings. Additionally, pregnant women, patients with a VL relapse or known comorbidities, patients on immunomodulatory treatment, and severely anaemic patients with a haemoglobin (Hb) level <5 g dL^−1^ were excluded from participation. Using these criteria, 21 rK39 ICT-confirmed symptomatic patients were enrolled.

Healthy controls from West Pokot were actively recruited in the households and villages of enrolled VL patients. Individuals were eligible if they reported to be healthy, showed no symptoms or signs of VL and had no history of previous VL infection. After providing informed consent, healthy volunteers were tested with rK39 ICT and a malaria rapid diagnostic test (SD Bioline Malaria Differential P.f/Pan Test, Standard Diagnostics Inc., Suwon, South Korea) to confirm their negative status for *Leishmania* and *Plasmodium*, respectively. In total, 15 healthy controls were recruited.

### Sample collection and processing

Participants’ age, sex and clinical features were registered and venous blood was sampled from all study subjects. For healthy controls, blood was collected once, immediately after enrolment in the field. VL cases were followed up during their 17-day antileishmanial treatment of daily intramuscular or intravenous injections with SSG (20 mg kg^−1^ body weight) in combination with intramuscular PM injections (15 mg kg^−1^ body weight) (Kenya Ministry of Health, 2017). Patient blood was collected at baseline before the start of treatment (D0), after the 7th treatment dose (D7) and after the 17th and final treatment dose (D17). For blood sampling, 6 mL was collected in an ethylenediaminetetraacetic acid (EDTA) anticoagulation tube and another 5 mL collected in a serum isolation tube following the tube manufacturer's instructions.

Splenomegaly, defined as a palpable spleen below the left costal margin, was assessed in healthy controls at enrolment and in VL patients at D0, D7 and D17. Additionally, haematological parameters of VL patients were measured in the collected EDTA blood with an Ac-T diff Hematology Analyzer (Beckman Coulter, Brea, CA, USA) at Kacheliba Sub-County Hospital. Measured parameters included counts of leukocytes, lymphocytes, granulocytes, monocytes, erythrocytes and thrombocytes, as well as Hb and haematocrit levels. Haematology values were not determined for healthy controls due to the time interval between sample collection at the household and arrival of these samples at Kacheliba Hospital.

Immediately after haematology analysis and serum isolation, EDTA blood and serum samples were stored at −20°C at Kacheliba Hospital. For laboratory analysis of parasite load in EDTA blood samples and cytokine concentrations in serum samples (see the following 2 sections below), whole blood and serum samples of all participants were transferred to the Laboratory for Experimental Parasitology at Amsterdam University Medical Centre (AUMC) in the Netherlands, where they were stored at −80°C until tested. For the sample export from Kenya, a material transfer agreement was signed by Amref Health Africa Headquarters, Nairobi, Kenya as providing institution and the Laboratory for Experimental Parasitology at AUMC, Amsterdam, the Netherlands as recipient. The material transfer agreement was approved by the Amref Health Africa Ethics and Scientific Review Committee.

### Real-time polymerase chain reaction analysis

Quantitative real-time polymerase chain reaction (PCR) (qPCR) was used to confirm VL diagnosis and quantify *Leishmania* parasite load in blood. Briefly, 50 μL of EDTA blood was used for automated DNA extraction with NucliSENS EMAG (bioMérieux, Marcy-l’Étoile, France). In total, 1.25 μL of isolated DNA elution was tested with a qPCR protocol targeting *Leishmania* kinetoplast DNA (kDNA) and human glyceraldehyde 3-phosphate dehydrogenase as DNA extraction control. The qPCR was run on a Bio-Rad CFX96 real-time PCR instrument; the reagent mix and reaction conditions have been described in detail elsewhere (Hagos *et al*., 2024). To quantify blood parasite load in tested patient samples, each qPCR run included a standard curve of DNA extractions from a 10-fold dilution series of *L. donovani* promastigote culture (strain WR352) diluted in EDTA blood. The qPCR output was processed using Bio-Rad CFX Maestro 2.0 software.

All DNA isolates were also tested with qPCR for *Plasmodium falciparum* genomic material to exclude a malaria (co-)infection, which is another common protozoan disease in the study area which may influence the observed cytokine responses (Mueller *et al*., 2014; van den Bogaart *et al*., 2014). In total, 2.5 μL DNA template was tested with a qPCR protocol targeting the *P. falciparum* 18s rRNA gene as has previously been described (Kattenberg *et al*., 2012). The qPCR was run on a Bio-Rad CFX96 machine, and the results were analysed with Bio-Rad CFX Maestro 2.0 software.

### Multiplex cytokine analysis

This study measured the serum concentrations of 9 cytokines: IFN-*γ*, IL-6, IL-10, IL-12p70, IL-17A, IL-22, IL-27 and TNF-*α* were chosen based on their central role in VL immunology, their possible association with protective immunity and/or their potential as a biomarker for treatment response based on previous studies (Pitta *et al*., 2009; Kip *et al*., 2015; Dos Santos *et al*., 2016; Rodrigues *et al*., 2016). Additionally, IL-5 was included as an infrequently studied cytokine in VL patients (Kurkjian *et al*., 2006; Elshafie *et al*., 2011). Serum cytokine concentrations were determined by means of a 9-plex magnetic bead immunoassay, using a customized ProcartaPlex Human Mix & Match Panel (Invitrogen, Thermo Fisher Scientific, Waltham, MA, USA). The assay was performed according to the manufacturer's instructions. In total, 25 μL of undiluted serum samples were tested together with a 7-point standard curve of 4-fold serial dilutions and a non-template control that were included on each assay plate. All samples, standards and controls were measured in duplicate. After the final incubation step in the plate preparation, plates were read with a Bio-Plex 200 system (Bio-Rad, Hercules, USA). A minimum of 50 beads per cytokine was measured. Using Bio-Plex Manager software (version 6.0 Bio-Rad, Hercules, USA), a 5-parameter logistic standard curve was generated for each analyte to calculate its concentration in the tested samples. The lowest detectable concentrations of the different cytokines (as calculated with the generated standard curves) were: 0.25 pg mL^−1^ for IFN-*γ*, 0.17 pg mL^−1^ for IL-5, 0.45 pg mL^−1^ for IL-6, 0.12 pg mL^−1^ for IL-10, 1.07 pg mL^−1^ for IL-12p70, 0.10 pg mL^−1^ for IL-17A, 0.68 pg mL^−1^ for IL-22, 0.99 pg mL^−1^ for IL-27 and 0.09 pg mL^−1^ for TNF-*α*.

### Statistical analysis

Both ProcartaPlex assay results and qPCR results were exported from the Bio-Rad software programs to Microsoft Excel data files. Together with the clinical and haematological data, these files were subsequently imported into statistical software IBM SPSS Statistics (version 28.0, Armonk, NY: IBM Corp.) for data processing and analysis. For all applied statistical tests, a *P* value <0.05 was considered significant.

Values for haematological parameters were compared with reference values for western Kenyan populations (Sing'oei *et al*., 2021). For normally distributed haematological variables, the mean and standard deviation (s.d.) were calculated; for skewed variables, including blood parasite loads, the median and interquartile range (IQR) were used.

Cytokine concentrations at D0 were compared between VL patients and healthy controls using the Mann–Whitney *U* test. To associate the measured cytokine profiles and blood parasite load at D0 with VL disease severity, VL patients were stratified into severe and non-severe cases. VL was considered severe when the patient met at least one of the measured clinical prognostic factors for mortality due to VL in East Africa, as reported by Abongomera *et al*. (2020): jaundice, oedema, bleeding, Hb ⩽ 6.5 g dL^−1^, body mass index (BMI) < 16 kg m^−2^ and splenic enlargement ⩾10 cm (Abongomera *et al*., 2020). Cytokine concentrations and parasite loads were compared between severe and non-severe VL patients using the Mann–Whitney *U* test. Correlations of D0 cytokine concentrations among themselves and with haematological parameters, *Leishmania* blood parasite loads and spleen size were calculated using Spearman's rank test. To statistically assess changes in cytokine concentrations, splenic enlargement and haematology parameters in VL patients between D0 and D17, a paired *t*-test was used for normally distributed variables and a Wilcoxon-signed rank test for non-normal variables.

## Results

### Participant characteristics

Of the 21 enrolled symptomatic patients with a positive result for rK39 ICT, 1 patient was negative for kDNA with qPCR and was therefore considered negative for VL. This meant that 20 patients with confirmed VL infection based on positive rK39 ICT and kDNA qPCR results were included in the final analysis. These patients are marked as VL01 to VL20 in the results described below. They had a median age of 20 years (IQR, 14–25) and 15 patients were male. All 15 healthy controls were negative for rK39 ICT, malaria rapid diagnostic test and kDNA qPCR; 1 control was positive for *P. falciparum* in qPCR and was therefore excluded from further analysis. The median age of the 14 included controls was 22 years (IQR, 9–30) and 7 controls were male.

The symptoms reported in VL patients at D0 are presented in Table 1. Nearly all patients presented with splenomegaly, weight loss and chills. Fifteen cases were classified with severe VL, presenting with Hb ⩽ 6.5 g dL^−1^ (*N* = 5), BMI < 16 kg m^−2^ (*N* = 6) and/or splenic enlargement ⩾10 cm (*N* = 12). Patients VL09 and VL15 met all these 3 criteria and were therefore considered most ill. Thirteen of the 20 VL patients were clinically followed up until the end of treatment; 7 patients were lost to follow-up due to voluntary drop-out before D17 or premature departure from the hospital after discharge. In followed-up patients, spleen enlargement decreased significantly (*P* = 0.016, Fig. S1) and all other symptoms resolved by D17 of treatment.

**Table 1.** Frequency of symptoms and signs reported at D0 among the study population of VL patients

Symptoms	D0 (*N* = 20^a^)
Splenomegaly	19^b^
*Splenic enlargement below the costal margin in cm (mean* *±* *s.d.)*	*10.0* *±* *4.1*^b^
Weight loss	19
*BMI in kg m^−2^ (median, IQR)*	*17.1 (14.5–18.6)*^c^
Chills	18
Headache	15
Malaise	14
Cough	13
Abdominal swelling	12
Fatigue	9
Sweating	8
Fever (forehead temperature >38°C)	6
Anorexia	5
Myalgia	4
Abdominal pains	4
Vomiting	3
Arthralgia	2
Palpitations	2
Shortness of breath	1

a Excluding 1 patient who was negative for VL in qPCR.

b Excluding 1 patient whose spleen size was not measured.

c Excluding 1 patient whose weight was not measured.

### Haematology

**Figure 1.**
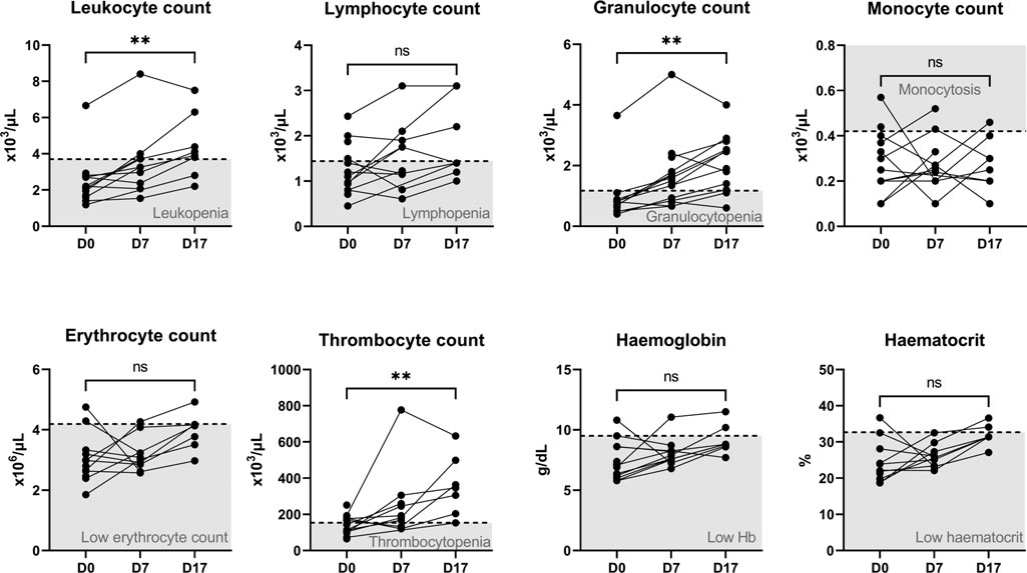
Haematological parameters in VL patients on SSG-PM treatment. Each line represents an individual VL patient. The shaded zones indicate the values below or above healthy reference ranges. The statistical significance of change between D0 and D17 is indicated above the plots. Wilcoxon-signed rank test was used for leukocyte count and haematocrit, all other parameters were tested with a paired *t*-test. **P < 0.01, ns: non-significant (*P* > 0.05).

The haematological profile of VL patients at D0 was characterized by pancytopenia (Fig. 1): out of 13 patients for whom haematology assessment was done, 12 had leukopenia (median, 2.20 × 10^3^ μL^−1^; IQR, 1.66–2.75 × 10^3^ μL^−1^), 9 had lymphopenia (mean, 1.24 × 10^3^ μL^−1^; s.d., 0.57 × 10^3^ μL^−1^) and 12 had low granulocyte counts (median, 0.70 × 10^3^ μL^−1^; IQR, 0.55–0.86 × 10^3^ μL^−1^). Anaemia was common, with a mean Hb level of 7.40 g dL^−1^ (s.d., 1.55 g dL^−1^) and a mean erythrocyte count of 3.03 × 10^6^ μL^−1^ (s.d., 0.81 × 10^6^ μL^−1^). Haematocrit was lowered in 11 of 12 measured VL patients (median, 22.10%; IQR, 20.16–27.10%). Seven of 12 patients with a measured thrombocyte count were thrombocytopenic (mean, 139.68 × 10^3^ μL^−1^; s.d., 55.02 × 10^3^ μL^−1^).

Haematology assessment at the end of treatment (D17) was performed for 8 VL patients (Fig. 1). They showed a statistically significant increase in leukocyte (*P* = 0.008), granulocyte (*P* = 0.008) and thrombocyte (*P* = 0.008) counts. Erythrocyte count, Hb and haematocrit exhibited a non-significant increasing trend.

### *Leishmania* parasite loads

**Figure 2.**
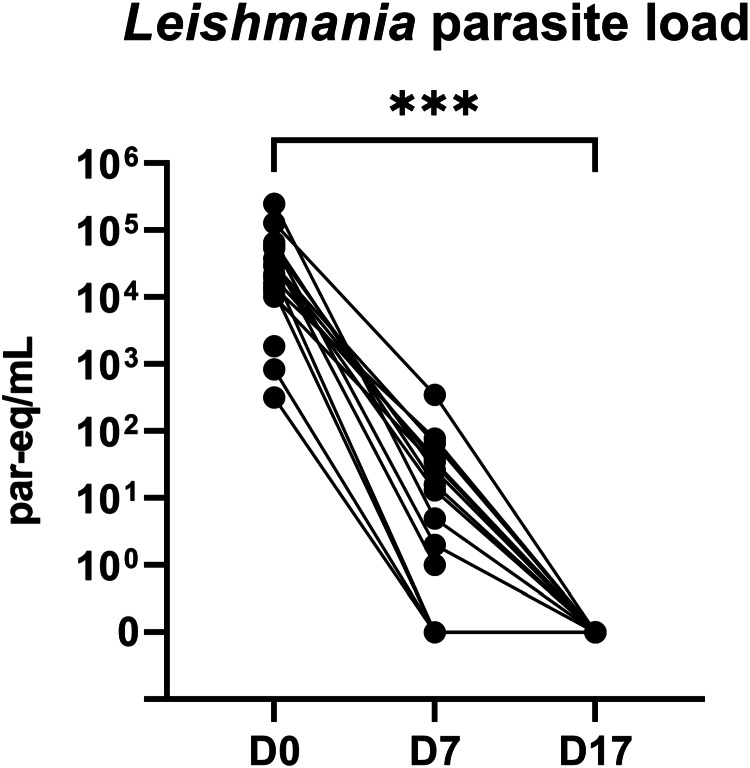
*Leishmania* parasite load in peripheral blood in VL patients on SSG-PM treatment. Parasite load was measured using kDNA qPCR. Each line represents an individual VL patient. Difference in load between D0 and D17 was calculated with Wilcoxon-signed rank tests. Par-eq: parasite equivalents; ***P < 0.001.

At baseline, VL patients had a median parasite load of 2.6 × 10^4^ parasite equivalents (par-eq) mL^−1^ blood (IQR, 1.3 × 10^4^–5.0 × 10^4^ par-eq mL^−1^). Blood parasite loads did not differ significantly between severe and non-severe VL patients and were not associated with haematological parameters (data not shown). For 15 patients, a D7 blood sample was collected and analysed with qPCR as well: they all showed a steep decline in parasite load (median, 13 par-eq mL^−1^; IQR, 0–40 par-eq mL^−1^, Fig. 2). A D17 blood sample was collected for 13 VL patients, which were all negative for kDNA at this time point.

### Cytokine levels at baseline (D0)

**Figure 3.**
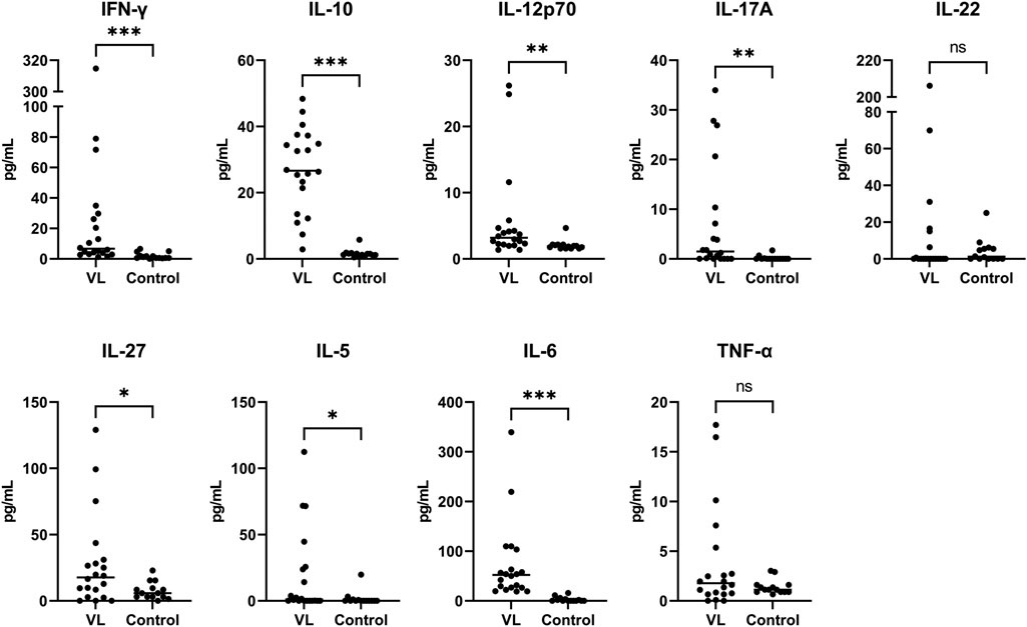
Serum cytokine concentrations in VL patients (at D0, before treatment initiation) and healthy controls. Horizontal line indicates the median value. Difference between VL cases and healthy controls was tested using a Mann–Whitney *U* test. *P < 0.05, **P < 0.01, ***P < 0.001, ns: non-significant (*P* > 0.05).

Serum cytokine concentrations at D0 were measured for the 20 qPCR-confirmed VL patients and 14 healthy controls (Fig. 3). Cytokine levels in healthy controls were generally low to undetectable, while VL cases showed significantly increased levels of IFN-*γ*, IL-5, IL-6, IL-10, IL-12p70, IL-17A and IL-27. Some cytokines were undetectable at D0 in multiple patients: most notably, 8 patients had undetectable IL-5, 6 patients had undetectable IL-17A and 12 patients had undetectable IL-22. Severe VL patients had significantly lower IFN-*γ* concentrations (*P* = 0.042) and significantly higher IL-10 concentrations (*P* = 0.016) than the non-severe VL patients (Fig. S2). The levels of the other cytokines did not differ significantly between these patient groups.

### Cytokine correlations at baseline (D0)

Several statistically significant correlations were found between cytokine concentrations on D0 (Table 2). Pro-inflammatory IFN-*γ* was negatively correlated with anti-inflammatory IL-10 and *Leishmania* parasite density in blood, while there was a positive correlation between IL-10 and parasite load. IL-17A and IL-5 correlated positively with each other and with IL-6, IL-12p70, IL-22, IL-27 and TNF-*α*. Furthermore, there was a positive correlation between IL-27 *vs* IL-6 and IL-12p70. No significant correlations were found between cytokine concentrations on D0 *vs* haematological parameters and spleen size (data not shown).

**Table 2.** Spearman's rank correlation coefficients between cytokine concentrations and blood parasite load on D0 in VL patients

	IFN-*γ*	IL-10	IL-12p70	IL-17A	IL-22	IL-27	IL-5	IL-6	TNF-*α*
IL-10	−0.59**								
IL-12p70	0.24	0.19							
IL-17A	0.36	0.07	0.75***						
IL-22	0.15	0.18	0.36	0.44*					
IL-27	0.37	0.10	0.75***	0.81***	0.33				
IL-5	0.17	0.36	0.72***	0.72**	0.56**	0.72**			
IL-6	0.46*	−0.15	0.45*	0.65**	0.19	0.51*	0.46*		
TNF-*α*	0.25	0.14	0.55**	0.78***	0.54*	0.56**	0.75**	0.62**	
Par. load	−0.62**	0.51*	−0.06	−0.06	−0.04	0.09	0.17	−0.26	−0.09

Par. load: *Leishmania* parasite load in blood, measured with qPCR.

*P < 0.05, **P < 0.01, ***P < 0.001.

### Cytokine levels during follow-up

For 12 of the 20 qPCR-confirmed VL cases, serum samples were also collected on D7 and D17 of treatment; 3 of the 20 VL patients only had a follow-up sample for D7, and 1 only had a follow-up sample for D17. For the remaining 4 VL patients, no follow-up samples were collected.

**Figure 4.**
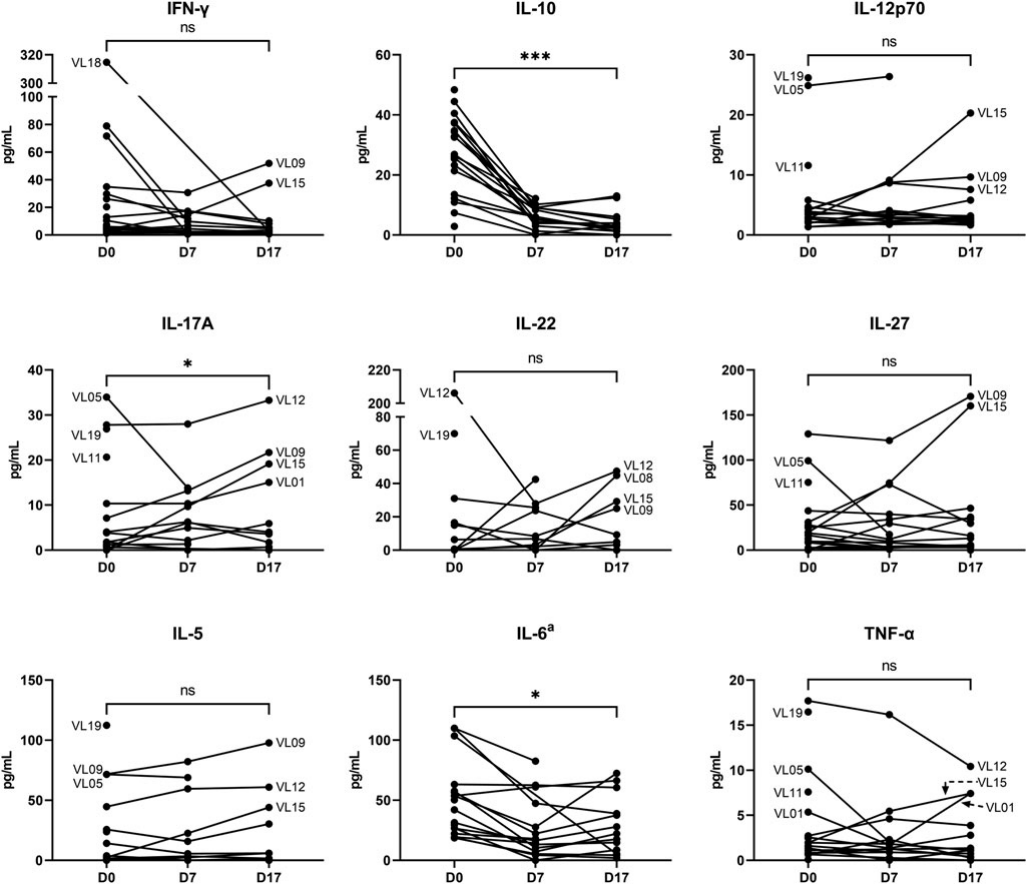
Changes in serum cytokine concentrations in VL patients on SSG-PM treatment. Each line represents an individual VL patient, outlying lines or points have been labelled with their corresponding patient number. The statistical significance of concentration change between D0 and D17, calculated with Wilcoxon-signed rank test, is indicated above the plots. ^a^Outlying D0 data points of 2 VL patients without follow-up are not shown. *P < 0.05, ***P < 0.001, ns: non-significant (*P* > 0.05).

At the end of treatment (D17), levels of IFN-*γ*, IL-5, IL-6, IL-10, IL-12p70, IL-17A and IL-27 were still significantly elevated compared to healthy controls (data not shown). Levels of IL-6 and IL-10 decreased significantly on D17 compared to pre-treatment concentrations, while IL-17A concentrations increased (Fig. 4). All other cytokines did not show statistically significant changes from D0 to D17.

However, when plotting the changes in cytokine concentrations in individual patients, several cytokines showed considerable inter-patient variation in their D0 concentrations and dynamics during treatment (Fig. 4). While most patients showed a decrease in IFN-*γ* concentration after treatment initiation, patients VL09 and VL15 showed an increase in this cytokine, with higher levels on D17 than on D0. These 2 patients exhibited a similar increasing trend for IL-5, IL-12p70, IL-17A, IL-22 and IL-27. Additionally, several patients had a relative increase in IL-6 between D7 and D17.

## Discussion

This was the first study in Kenya to measure immunological, parasitological and haematological parameters in blood of VL patients before, during and after SSG-PM treatment of their infection. In untreated patients, various inflammatory and regulatory cytokines were elevated in serum, and haematology was characterized by pancytopenia. Blood parasite levels became undetectable after treatment, clinical and haematological parameters improved, and cytokine concentrations generally decreased. However, IL-17A levels increased, suggesting continuation of inflammatory processes during disease resolution.

Splenomegaly and pancytopenic peripheral blood counts in the participating VL patients are a hallmark of this disease, and a result of chronic splenic and systemic inflammation as well as myelodysplastic processes in the infected spleen (Poulaki *et al*., 2021; Costa *et al*., 2023; Debash *et al*., 2023). A significant number of patients showed severe disease, presenting with extreme splenomegaly, underweight and low Hb. Spleen size significantly decreased during treatment and leukocyte, granulocyte and thrombocyte counts were restored, but most patients assessed at D17 were still anaemic. These haematological findings have also been reported in Ethiopian VL patients and are in line with previous studies showing that it may take between 30 and 60 days post-treatment initiation for Hb levels to fully recover (Goto *et al*., 2017; Mulaw *et al*., 2018; Shiferaw *et al*., 2021). Nevertheless, the resolution of symptoms and reconstitution of multiple haematological values after SSG-PM treatment suggest that this therapy was effective in the study cohort. This is in line with a previous pharmacovigilance trial conducted in Kenya in 2012 and 2013, where SSG-PM treatment resulted in initial cure in 99.4% of the treated patients (Kimutai *et al*., 2017).

Treatment effectiveness was also implied by monitoring *Leishmania* parasite load in blood: kDNA concentrations in blood decreased during treatment and were undetectable in all patients on D17. A future relapse thus seemed unlikely, as a previous study demonstrated that relapsing patients often have detectable circulating parasite DNA at the end of initial treatment (Verrest *et al*., 2021). Nevertheless, live parasites could potentially remain in infected tissues despite a negative qPCR result on blood (Cascio *et al*., 2002; Aquino *et al*., 2023). Therefore, late treatment failure could not be completely excluded, as the participants were not followed up after D17 and earlier studies in East Africa reported VL relapse rates of 2.4% and 3.6% after SSG-PM treatment (Kimutai *et al*., 2017; Musa *et al*., 2023).

The immune response to VL has frequently been characterized by the release of a multitude of pro-inflammatory and regulatory cytokines, which shape the disease pathogenesis and clinical presentation (Dayakar *et al*., 2019; Costa *et al*., 2023). In this study, 7 of 9 analysed cytokines were elevated in VL patients at D0 compared to non-infected controls, with the most pronounced increases in IFN-*γ*, IL-10, IL-6 and IL-27. IFN-*γ* is one of the key cytokines involved in the activation of infected macrophages, required for an adequate anti-parasitic immune response. Many other studies in untreated VL patients also found high IFN-*γ* levels as well as elevated IL-12p70, which is produced by antigen-presenting cells (APCs) to stimulate IFN-*γ* production by T helper-1 (Th1) cells (Ansari *et al*., 2006; Duthie *et al*., 2014; van den Bogaart *et al*., 2014; Dos Santos *et al*., 2016; Tadesse *et al*., 2021). This implies that *Leishmania* parasites mainly modulate the IFN-*γ* signalling pathway, rather than its secretion, in order to survive inside phagocytic cells. Crucial in the inhibition of IFN-*γ* effector functions is IL-10 (Nylén and Sacks, 2007). This anti-inflammatory cytokine induces various processes that favour parasite survival, such as downregulation of major histocompatibility complex II expression and IL-12p70 production by APCs, leading to a decreased activation of *Leishmania*-specific Th1 cells and lower IFN-*γ* production (Nylén and Sacks, 2007; Singh *et al*., 2012). These mechanisms were also reflected in the results of this work, where pre-treatment concentrations of IL-10 and IFN-*γ* were negatively correlated. The lower IFN-*γ* and higher IL-10 levels in severe VL patients, and the negative correlation between IFN-*γ* concentration and blood parasite load on D0, underline the pivotal role of IFN-*γ* in suppressing parasite replication and mitigating disease severity.

IL-6 is a pleiotropic cytokine with a multitude of possible effector functions in VL infection, while IL-27 is mainly known for its anti-inflammatory properties (Yoshida and Hunter, 2015; Dayakar *et al*., 2019). This study observed elevated serum concentrations of IL-6 and IL-27 in untreated VL patients, as well as a strong correlation between these cytokines. Both findings have also been reported by studies in Brazil, where high levels of IL-6 and IL-27 were associated with severe VL disease and increased fatality (Costa *et al*., 2013; Dos Santos *et al*., 2016). However, in the current study, pretreatment concentrations of IL-6 and IL-27 were not significantly different between severe and non-severe VL patients. This may reflect differences in VL immunology between *L. infantum-*infected patients from Brazil and patients from Kenya, which is endemic for *L. donovani*.

Several VL patients exhibited increased levels of IL-17A at baseline, similar to what has previously been observed in studies from Sudan and Brazil (Duthie *et al*., 2014; Nascimento *et al*., 2014; van den Bogaart *et al*., 2014). This cytokine has pro-inflammatory effects and is involved in the recruitment of neutrophils as well as the induction of IFN-*γ* secretion (Gonçalves-de-Albuquerque *et al*., 2017). Mouse studies have proposed that these effector functions are important for parasite control in the spleen and liver, but may also contribute to damaging inflammation and hepatosplenomegaly (Ghosh *et al*., 2012; Nascimento *et al*., 2014). In humans, production of IL-17A and IL-22 has been associated with resistance to symptomatic *L. donovani* infection (Pitta *et al*., 2009). However, increase in serum IL-17A in VL patients enrolled in the current study did not protect them from developing clinical disease. Possibly, their IL-17A response was insufficient to confer VL resistance, which is likely to rely on other host factors as well. As for IL-22, a Th17 cytokine which is generally associated with epithelial immunity (Dudakov *et al*., 2015), this was the first study to measure its concentration in active, untreated VL patients. There was considerable variation in IL-22 levels between patients both on D0 and D17; the equivocal role of IL-22 in VL immunology therefore needs to be investigated further.

The T-helper 2 (Th2) cytokine IL-5 is mainly known for its eosinophil activating effect, for example in the mucosal immune response to intestinal helminths (Maizels and Balic, 2004). Its role in VL immunology is generally considered to be negligible. Nonetheless, in this study IL-5 was unexpectedly increased both before and after treatment in several VL patients compared to controls. It is possible that this was the result of an underlying comorbidity, as participants were not screened for helminth infections. However, IL-5 could play some role in the immune response to *Leishmania* as well: a previous study in Sudan also reported increased IL-5 concentrations in VL patient sera compared to healthy controls from the same region, together with increased eosinophil activation (Elshafie *et al*., 2011). All in all, these observations suggest the need for increased attention for IL-5 in future immunological studies.

By measuring cytokine concentrations during and at the end of VL treatment, this study has provided more insight into the possible effect of SSG-PM therapy on the VL immune response. A diminishing inflammatory response was implied by the decreasing (though non-significant) trend in IFN-*γ* concentrations in the treated VL patients, which has been reported before (Cenini *et al*., 1993; Caldas *et al*., 2005; Ansari *et al*., 2007; Duthie *et al*., 2014). Simultaneously, IL-10 concentrations decreased in all patients during treatment, indicating that IL-10-mediated immune suppression is reduced when parasites are cleared. On the other hand, IL-17A levels showed a significant increase over the course of treatment and there was a resurgence of IL-6 in several VL patients between D7 and D17. IL-6 is essential for the differentiation of Th17 cells, which are a major source of IL-17A (Korn *et al*., 2009). In turn, IL-17A may induce the production of IL-6 (Korn *et al*., 2009). Continued elevation of IL-17A and IL-6 after VL treatment has been reported previously, and may be the result of deregulation by IL-10 as well as an increase in parasitic antigens derived from dead amastigotes that trigger these immune responses (Ansari *et al*., 2006, 2007; Nascimento *et al*., 2014). Alternatively, IL-17A and IL-6 may play a role in the resolution of VL through IL-17A-induced neutrophil activation and a switch in IL-6 effector function, from suppression to maintenance of the Th1 response (Ansari *et al*., 2006; Pitta *et al*., 2009).

Patients VL09 and VL15 had deviant results for multiple measured variables. They presented with the most severe symptoms at D0, and they showed an aberrant increase in the concentrations of IFN-*γ*, IL-5, IL-12p70, IL-27 and TNF-*α* levels during treatment. Additionally, VL09 and VL15 had some of the highest IL-6 and IL-17A concentrations on D17. These results suggest an increase in inflammatory response upon treatment, involving Th1, Th2 and Th17 cytokines. As speculated above, the release of high levels of killed parasites from tissues into the circulation may have led to temporary immune activation in VL09 and VL15, which is supported by the strong increase in leukocyte counts in VL15 on D17. This prolonged inflammation could have detrimental effects on the host as well. Indeed, patient VL09 had a decreasing but unresolved splenomegaly on D17 (spleen size at the end of treatment was not measured for VL15).

A limitation of this study was the loss to follow-up of 7 participants, meaning that there remains some uncertainty regarding the efficacy of SSG-PM in the complete cohort. Furthermore, there was no post-treatment monitoring due to the exploratory set-up. The findings regarding cytokine dynamics upon SSG-PM treatment should therefore be confirmed by future investigations with an extended follow-up to assess definitive cure. Such studies may also further evaluate the use of cytokines as prognostic markers for long-term treatment outcome. Based on the results of this work, interesting candidates could be IL-10, IL-6 and IL-17A. Ideally, future studies should screen for more potential co-infections besides malaria, such as HIV and helminths, which could influence the VL immune response. Due to resource constraints, this fell outside the scope of the current research. However, HIV co-infections in VL patients are rare in West Pokot (Mueller et al., 2014), and chances of uneven distribution of comorbidities among cases and controls were reduced by their household- and village-matching, which also diminished confounding by genetic and environmental factors.

In conclusion, SSG-PM treatment of VL patients in Kenya relieved symptoms, eliminated *L. donovani* parasites from the circulation and generally reduced the levels of inflammatory and regulatory cytokines. The increase in IL-17A during treatment could point towards a potential role of this cytokine in parasite clearance, which may be supported by IL-6. A number of findings warrant further investigation, including the variability in IL-22 response and the possible role for IL-5 in immunity to VL. Moreover, longitudinal cohort studies are required to elucidate the use of serum cytokine levels before and after treatment as a biomarker for therapeutic efficacy.

## Supporting information

van Dijk et al. supplementary material van Dijk et al. supplementary material

## Data Availability

The datasets used in this study are available from the corresponding author upon reasonable request.

## Supplementary material

The supplementary material for this article can be found at https://doi.org/10.1017/S0031182024000751.
