# Supplementary material for: Cytokine profiles, blood parasite load and clinical features of visceral leishmaniasis in West Pokot County, Kenya
Source: Parasitology. 2024 Sep 23;151(7):753–61. doi: 10.1017/S0031182024000751 (PMC11474019; doi:10.1017/S0031182024000751)
Supplement: van Dijk et al. supplementary material [file S0031182024000751sup001.docx]

**Cytokines profiles in visceral leishmaniasis in Kenya - Supplementary files**

**
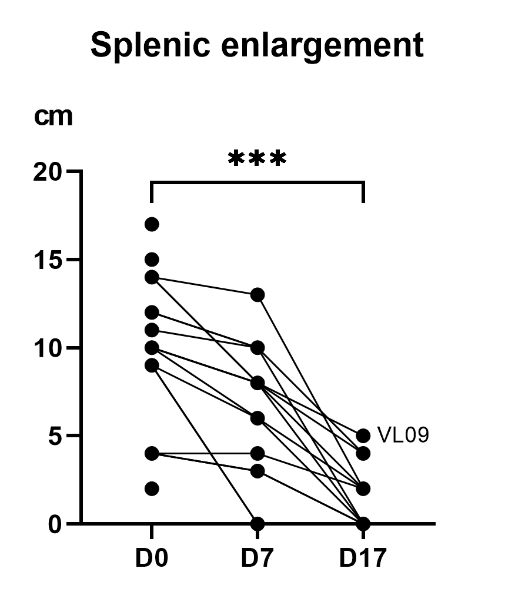
**

**S1 Figure: Splenic enlargement in VL patients on SSG-PM treatment.** Measured below the left costal margin. Each line represents an individual VL patient (D17 value for patient VL09 has been labelled). The statistical significance of change between D0 and D17, calculated with Wilcoxon signed rank test, is indicated above the plot. ***p<0.001.

**
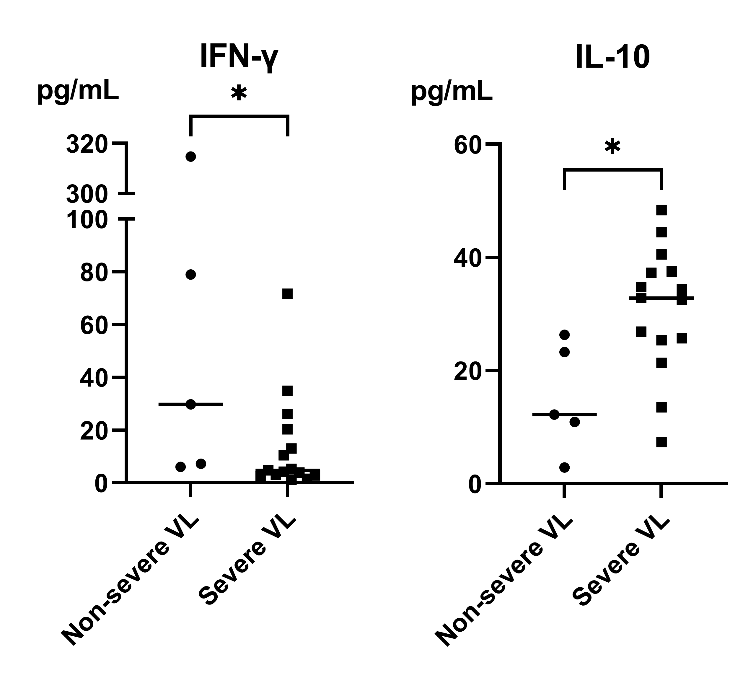
**

**S2 Figure: Serum concentrations of IFN-γ and IL-10 in non-severe and severe VL patients at D0.** Horizontal line indicates the median value. Difference between non-severe and severe cases was tested using a Mann-Whitney U test. *p<0.05.
